# Denaturation of proteins by surfactants studied by the Taylor dispersion analysis

**DOI:** 10.1371/journal.pone.0175838

**Published:** 2017-04-20

**Authors:** Aldona Jelińska, Anna Zagożdżon, Marcin Górecki, Agnieszka Wisniewska, Jadwiga Frelek, Robert Holyst

**Affiliations:** 1 Institute of Physical Chemistry, Polish Academy of Sciences, Warsaw, Poland; 2 Institute of Organic Chemistry, Polish Academy of Sciences, Warsaw, Poland; Russian Academy of Medical Sciences, RUSSIAN FEDERATION

## Abstract

We showed that the Taylor Dispersion Analysis (TDA) is a fast and easy to use method for the study of denaturation proteins. We applied TDA to study denaturation of β-lactoglobulin, transferrin, and human insulin by anionic surfactant sodium dodecyl sulfate (SDS). A series of measurements at constant protein concentration (for transferrin was 1.9 x 10^−5^ M, for β- lactoglobulin was 7.6 x 10^−5^ M, and for insulin was 1.2 x 10^−4^ M) and varying SDS concentrations were carried out in the phosphate-buffered saline (PBS). The structural changes were analyzed based on the diffusion coefficients of the complexes formed at various surfactant concentrations. The concentration of surfactant was varied in the range from 1.2 x 10^−4^ M to 8.7 x 10^−2^ M. We determined the minimum concentration of the surfactant necessary to change the native conformation of the proteins. The minimal concentration of SDS for β-lactoglobulin and transferrin was 4.3 x 10^−4^ M and for insulin 2.3 x 10^−4^ M. To evaluate the TDA as a novel method for studying denaturation of proteins we also applied other methods i.e. electronic circular dichroism (ECD) and dynamic light scattering (DLS) to study the same phenomenon. The results obtained using these methods were in agreement with the results from TDA.

## Introduction

Proteins and surfactants are commonly used in the pharmaceutical, food, and cosmetics industries [1,2]. Therefore, interactions between them are intensively studied [3–8]. The addition of surfactants to protein solutions changes the physical properties of the protein i.e. unfolds proteins and causes aggregation and adsorption of proteins to surfaces [9,10]. Both the structural stability of the protein and the molecular structure of the surfactant (charge, length, shape of the polar and apolar part of the surfactant) has an impact on their mutual binding affinity. The nature of the interactions of proteins with ionic surfactants is both electrostatic and hydrophobic. When ionic surfactants are added to protein solution, the monomers of surfactant bind electrostatically to the oppositely charged residues on the protein. Therefore, the strength of interactions depends on the net charge of protein and surfactant. If the surfactant is negatively charged and the net charge of the protein is positive, then the precipitation of the complex takes place [11], due to neutralization of the charge on protein. This precipitated complex can be redissolved by adding an excess of surfactant. Interactions between negatively charged protein and anionic surfactant do not lead to the appearance of precipitation because the protein-anionic surfactant complex is always negatively charged. In contrast to ionic surfactants, nonionic surfactants weakly interact with proteins due to the lack of the contribution of electrostatic forces [12]. Nonionic surfactants bind to protein through hydrophobic interactions and hydrogen bonds. These interactions do not influence strongly the structure of protein. The protein-surfactant complexes have been examined using circular dichroism spectroscopy (CD) [13–15], nuclear magnetic resonance (NMR) [15], dynamic light scattering (DLS) [16–18], fluorescence correlation spectroscopy (FCS) [19–21], small angle X-ray scattering (SAXS) [22,23] and small angle neutron scattering (SANS) [24]. ECD allows studying structural changes at the level of secondary and tertiary structure. NMR is a good method to compare the conformational changes of protein under different experimental conditions. DLS and FCS are often used to measure the hydrodynamic radii of the protein-surfactant complexes. Small angle X-ray scattering (SAXS) and small angle neutron scattering (SANS) give information about the shape and structure of the complexes.

Here, we present application of TDA to study denaturation of β-lactoglobulin, transferrin and insulin under the influence of anionic surfactant—sodium dodecyl sulfate (SDS). We show that the Taylor dispersion analysis [25–30], a simple method applicable at chromatographic equipment, can be used for rapid determination (at the time scale of minutes) of denaturation of proteins. Chromatographic equipment which is commonly used in research laboratories. In the paper we present application of TDA to study denaturation of β-lactoglobulin, transferrin and insulin under the influence of anionic surfactant—sodium dodecyl sulfate (SDS). Additionally, other methods such as electronic circular dichroism and dynamic light scattering are applied for verifying the results obtained by the Taylor dispersion method.

## Materials and methods

### Materials

β-lactoglobulin, transferrin, and insulin were purchased from Sigma-Aldrich Chemie (Steinheim, Germany). Anionic surfactant sodium dodecyl sulfate (SDS) was purchased from Carl Roth GmbH + Co. (Karlsruhe, Germany). All compounds were dissolved in phosphate-buffered saline (PBS). One tablet dissolved in 200 mL of deionized water yields 0.01 M phosphate buffer, 0.0027 M potassium chloride and 0.137 M sodium chloride, pH 7.4, at 25°C. The tablets of buffer were purchased from Sigma-Aldrich Chemie (Steinheim, Germany).

### Apparatus

The experiments were carried out using an apparatus from Shimadzu Corporation (Kyoto, Japan). The equipment consists of the pump (LC-20 AD), degasser unit (DGU-20 A3R), autosampler (SIL-20AHT), capillary made of PEEK (polyether ether ketone). The capillary was 30 m long with a 0.25 mm inner diameter and coiled (the radius of curvature was 8 cm). The capillary was placed in the column oven (CTO- 20AC) and thermostated at 25 0.1°C. The absorbance was measured by UV-Vis (SPD-20A) detector connected to a PC computer using LC solution, version 1.25. Additionally, heating controller (ESM-3711-H bought from Laboplay, Poland) was used for keeping the proper temperature of a carrier phase. The carrier phase was transported through the capillary at the velocity of 31 cm s^-1^. The injection volume for all experiments was 10 μL.

### Taylor dispersion analysis (TDA)

The Taylor dispersion method is a fast and simple method for determination of diffusion coefficient. In this method an analyte is injected into the carrier phase which moves by the Poiseuille laminar flow through a long, thin and coiled capillary. The zone of the analyte widens as a result of diffusion and convection. The final concentration distribution (at the capillary end) is mapped by measurement of absorbance as a function of time. The diffusion coefficient is determined from the width of the concentration distribution at high flow rates (up to 31 cm/s) and with the use of the scaling equation:

$$D=-\;\frac{1}{48}\;\frac{u^{2}\;R^{2}}{\sigma_{\text{c}}A\cdot \text{Lambert W}\left(-1,-\;\frac{1}{192}\;\frac{r\;{\gamma \text{ e}}^{-\;\frac{\text{B}}{\text{A}}}}{R\;\rho \;\sigma_{\text{c}}\text{A}}\right)}$$ (1)

Where: σ_c_ is the dispersion coefficient in a coiled capillary, ρ is the density of the carrier phase, γ is the viscosity of carrier phase, R is the internal capillary radius, r is the external radius of curvature of the coiled capillary, A = 0.87 ± 0.02 and B = -3.8 ± 0.2 are fitted parameters. The formula 3.2.1 can be simplified by replacement of the function LambertW (-1,*x*) with its asymptotic expansion:

$$\text{Lambert W}\;\left(\;-1,x\right)\approx \text{W}\left(x\right)={\text{L}}_{1}-{\text{L}}_{2}+\frac{{\text{L}}_{2}}{{\text{L}}_{1}}+\frac{{\text{L}}_{2}\left(-2+{\text{L}}_{2}\right)}{2{\text{L}}_{1}^{2}}$$ (2)

Where: L_1_ = ln(-*x*) and L_2_ = ln (-ln (-*x*)).

Here, we apply modified Taylor dispersion method which is more accurate and faster than classical Taylor’s method. The details of this modification are described in our previous publication [25].

### Electronic circular dichroism (ECD)

ECD spectra were recorded on a Jasco J-815 spectrometer (Jasco Corporation, Tokyo, Japan) equipped with the 150 W xenon lamp in the range of 190–340 nm using quartz cell of 0.01, 0.1 and 1 cm path length at room temperature. All spectra reported here (both for protein and protein with some amount of SDS) were recorded using a standard sensitivity, a scanning speed of 50 nm min^-1^, a step size of 0.1 nm, a bandwidth of 1 nm, a response time of 1 s, and an accumulation of 5 scans. Baseline correction was achieved by subtracting the spectrum of phosphate-buffered saline (PBS) as a solvent recorded under the same conditions. The reported mean residue ellipticity values were expressed in the unit of deg cm^2^ dmol^-1^. They were obtained using a molecular mass of each relevant protein and a total number of amino acids. For estimation of the secondary structural composition the ECD spectra were submitted to the Jasco Secondary Structure Estimation (SSE) software based on the Principle Component Regression method. The multivariate analysis allowed us to obtain quantitative data of helix, sheet and random coil contents from ECD spectra.

### Dynamic light scattering

The dynamic light scattering experiments were conducted using Brookhaven Instruments BI-200SM Research Goniometer System with laser wavelength 514 nm. All DLS measurements were prepared in 298 K and scattering angles 40, 60, 80, 90, 120 and 150 degrees. The time-depended fluctuation in the scattering intensity *I*(*q*,*t*) was measured. The diffusion coefficient was extracted from the decay time of the autocorrelation function *g*_*2*_(q,τ):

$$g_{2}\left(q,\tau \right)=\frac{<I\left(q,t\right)\cdot I\left(q,t+\tau \right)>}{<I\left(q,t\right)><I(q,t>}\;$$ (3)

where: q is wavevector and τ is the time difference. The correlation function was fitted by one or two exponential functions. The samples were filtered through a syringe filter with a diameter of pores 0.2 μm, centrifuged at 2000 rpm speed and thermostated for 10 minutes. We collected the data for 15 minutes for each angle. Because the amplitude of autocorrelation function was low the effect of heterodyne was observed. The signal coming from the sample and detected at the photodetector was in this case a mixture from our proteins/surfactants and from the solvent i.e. dynamic (correlated) scattered light and the static (uncorrelated at studied time scale) scattered light. In consequence of small sizes of micelles and proteins and their small concentration the level of correlated signal was very low. An influence of the static light source would be negligible if the intensity of the normalized autocorrelation function *g*_2_(*q*,0) was higher than 0.7. In our case it was between 0.4 and 0.5. Thus we had to analyze the signal taking into account both components in the scattered light and use the heterodyne method. According to Geissler [31] we corrected the standard formula (see Eq 4). In this way we removed the uncorrelated signal from the autocorrelation function.

$$g_{2}\left(q,t\right)-1=2R\left(1-R\right)g_{1}\left(q,t\right)+R^{2}g_{1}{}^{2}\left(q,t\right)$$ (4)

Where *g*_2_(*t*) is an autocorrelation function of scattered intensities obtained in our experiment,*R* is a homodyne fraction, (1 − R) is a heterodyne fraction, and *g*_1_(*t*) is an autocorrelation function of electric field. For R = 1 (Eq 4) reduces to the standard formula.

## Results and discussion

**Fig 1:**
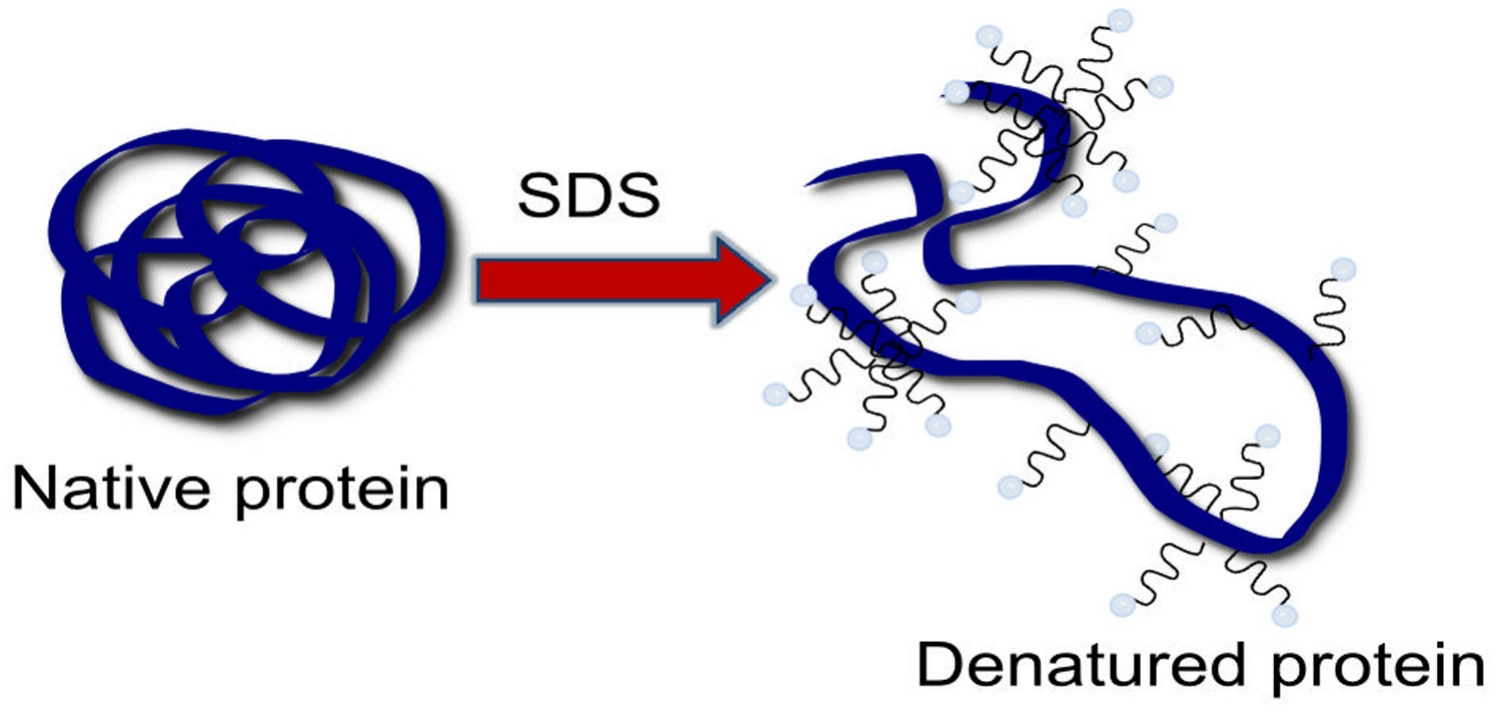
Schematic description of denaturation of proteins by surfactant.

**Fig 2:**
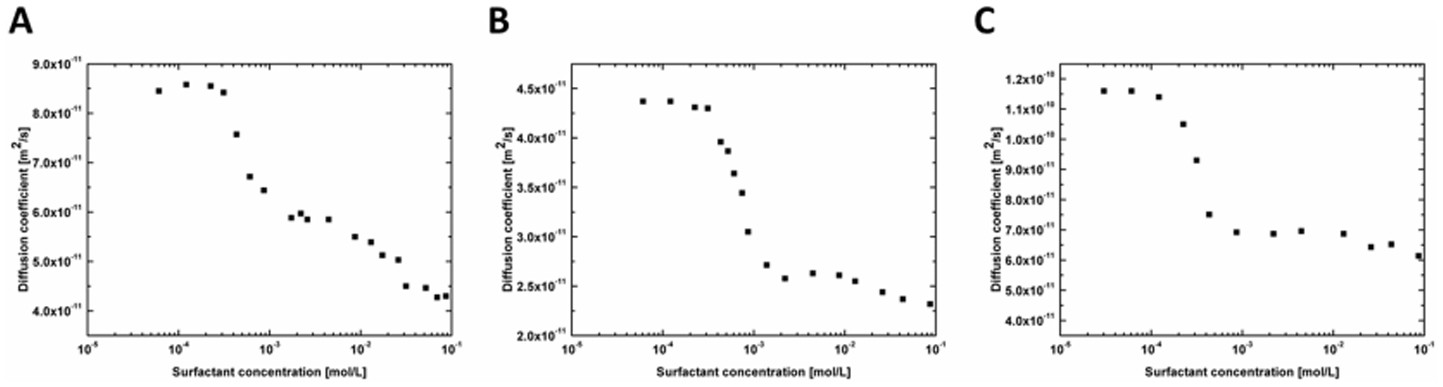
The diffusion coefficient for the β-lactoglobulin-SDS (A), transferrin-SDS (B) and human insulin-SDS (C) complexes as a function of SDS concentration determined using the Taylor dispersion analysis.

Denaturation process leads to unfolding of proteins and thus increases their size (Fig 1). According to the Stokes—Sutherland—Einstein equation an increase in the size of the object leads to a decrease of its diffusion coefficient. Using the Taylor dispersion method we can analyze denaturation of proteins on the basis of the changes in the value of the diffusion coefficient of proteins for different surfactant concentrations. The dependences of the diffusion coefficient on SDS concentration for transferrin-SDS, β-lactoglobulin-SDS and insulin-SDS systems are shown in the Fig 2. As expected, the diffusion coefficient of the protein-SDS complexes decreases with increasing SDS concentration till the tertiary structure of the protein is fully unfolded. We determined the minimum concentration of SDS initiating the changes in the tertiary structure of studied proteins. For β-lactoglobulin and transferrin the minimum concentration was 4.3 x 10^−4^ M and for insulin 2.3 x 10^−4^ M.

### Electronic circular dichroism measurements

We tested independently the sensitivity of TDA by application of the Electronic Circular Dichroism (ECD) spectroscopy to the same systems. ECD spectroscopy is commonly and effectively used to analyze the structures of protein. This method allows to study protein conformations and their changes due to external perturbations such as e.g. temperature, pressure, pH, denaturants, salts, and organic solvents [32–36].

Structural changes of proteins are observable by ECD due to the presence of intrinsic chromophores (aromatic amino acids) in proteins i.e. tryptophan, tyrosine. They give rise to signals in the near UV. When protein denatures, the ellipticity of a protein alters and the changes are proportional to the changes in the concentration of native and denatured forms.

**Fig 3:**
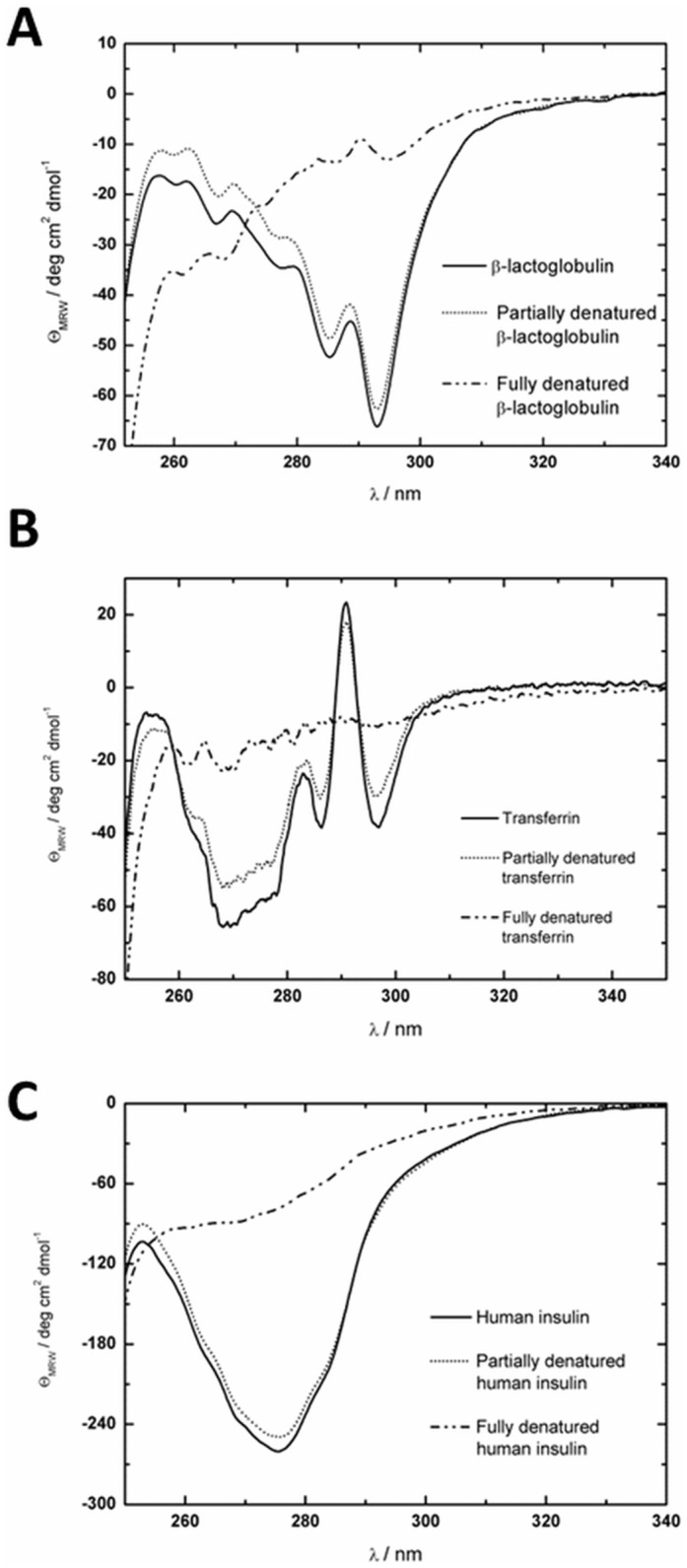
ECD spectra showing changes in the tertiary structure of β-lactoglobulin (A), transferrin (B) and human insulin (C) with increasing surfactant concentration. For β-lactoglobulin and transferrin the concentrations of SDS were 4.3 x 10^−4^ M and 8.7 x 10^−2^ M for partially and fully denatured protein, respectively. For insulin the concentrations of SDS were 2.3 x 10^−4^ M and 8.7 x 10^−2^ M for partially and fully denatured protein, respectively.

We measured the spectra of proteins characteristic for both the secondary (190–260 nm) and tertiary (250–340 nm) structure spectral range. Next we added SDS (denaturants) at various concentrations and repeated the measurements. Finally, the resulting ECD signals were used (see S1 Supporting Information for details) to find the minimum SDS concentrations which introduced changes in comparison to the native protein ECD spectrum. In the Fig 3 the ECD spectra in the range 250–340 nm for studied systems are shown.

The minimum concentration of SDS at which ellipticity starts to change is 4.3 x 10^−4^ M for β-lactoglobulin and transferrin and for insulin it is 2.3 x 10^−4^ M. The same results were obtained by TDA. After exceeding the critical concentration, the tertiary structure was fully destroyed and individual Cotton effects in the ECD spectrum disappeared. The details of ECD spectra for higher concentrations and the ECD spectra in the 190–260 nm range and their analysis are included in the S1 Supporting Information.

### Dynamic light scattering measurements

The dynamic light scattering method was used to obtain critical micelle concentration (CMC) of SDS in the solvent. A large number of probes with various concentration were tested and the first micelles were observed at the concentration (CMC) equal to 4.47 x 10^−3^ M.

We also applied dynamic light scattering to examine the structural changes of proteins after addition of SDS. We analyzed two types of samples: one containing only protein in a buffer and the other which contained both protein and SDS. The concentration of SDS was 8.67 x 10^−2^ M and it was the highest concentration as was used in earlier experiments using TDA and ECD. The autocorrelation functions obtained for samples containing surfactant are shown in S12 Fig (SI).

Data for β-lactoglobulin-SDS and transferrin-SDS systems were fitted using double-exponential fit (Figure A and B in S12 Fig) due to the presence of two species in solution: protein-SDS complexes and free micelles. The ratio of amplitudes of different components in the autocorrelation function was used to determine the ratio of the protein and micelles (Table 1). According to the Rayleigh's approximation, the intensity of scattered light is proportional to the diameter of the particle to the power of 6. Knowing this dependence, we could establish the amount of particles of different sizes.

**Table 1:** The ratio of the number of micelles to the number of proteins.

The ratio of amount of particles	
Micelles: denatured transferrin	7464: 1
Micelles: denatured β-lactoglobulin	105: 1

For insulin, we observed only one object in spite of the presence of two species, because of their similar size. In this case, dynamic light scattering is not sensitive enough to recognize these objects. Therefore, data for the insulin-SDS system were fitted using mono-exponential fit (Figure C in S12 Fig).

The diffusion coefficient of the β-lactoglobulin-SDS complex decreases from 7.44 x 10^−11^ m^2^/s at [SDS] = 0 to 4.34 x 10^−11^ m^2^/s at [SDS] = 8.67 x 10^−2^ M. The diffusion coefficient of the transferrin-SDS decreases from 4.95 x 10^−11^ m^2^/s at [SDS] = 0 to 2.06 x 10^−11^ m^2^/s at [SDS] = 8.67 x 10^−2^ M. The results indicate that diffusion coefficient of denatured protein is about two times lower than diffusion coefficient of native proteins. The comparison of diffusion coefficients determined using dynamic light scattering and Taylor dispersion analysis are shown in Table 2. The results obtained using these two methods are in accordance with each other. Small differences in the diffusion coefficient of native proteins are only observed. The reason for these differences is probably a sensitivity of dynamic light scattering to the presence of trace amount of partially denatured proteins in the samples. We note that the Taylor dispersion analysis is more sensitive to the overall change of structure of proteins.

**Table 2:** Comparison of diffusion coefficients determined using the Taylor dispersion analysis and dynamic light scattering before and after addition of SDS at high concentration to the solution.

Protein	D (TDA) ± SD x 10^−11^ (m^2^ s^-1^)	D (DLS) ± SD x 10^−11^ (m^2^ s^-1^)	D (TDA) ± SD x 10^−11^ (m^2^ s^-1^)	D (DLS) ± SD x 10^−11^ (m^2^ s^-1^)
Without addition of SDS			After structural transition	
**Transferrin**	4.37±0.01	4.54±0.06	2.16±0.08	1.93±0.06
**β-lactoglobulin**	8.14±0.01	6.98±0.05	4.29±0.01	4.20±0.22

## Conclusions

This study has shown that Taylor dispersion analysis is a convenient tool for tracking denaturation process of proteins. A single measurement takes only few minutes and is performed on a standard, commonly used chromatographic equipment The denaturation of three model proteins β-lactoglobulin, transferrin and insulin under the influence of anionic surfactant SDS was studied using this technique. Obtained results were confirmed using electronic circular dichroism and dynamic light scattering. Analysis of denaturation process by the Taylor dispersion method showed that diffusion coefficient decreases with increasing concentration of the surfactant in the sample. The minimum concentration of surfactant which induces structural changes was 4.3 x 10^−4^ M for β-lactoglobulin and transferrin, and for insulin 2.3 x 10^−4^ M. The same concentrations were obtained using electronic circular dichroism, and thus, the results from the Taylor dispersion method have been independently verified by other methods. Moreover, we observe by TDA and DLS that the diffusion coefficient of the denatured proteins is about two times smaller in comparison to the diffusion coefficient of native proteins. These findings are expected to be useful to understand the influence of surfactant on protein functionality.

## Supporting information

S1 Supporting Information (DOCX)

S1 Table Summary of ECD experiments for proteins under investigation. (TIF)

S1 Fig Changes in the tertiary structure of β-lactoglobulin with increasing surfactant concentration as shown by electronic circular dichroism. (TIF)

S2 Fig Changes in the tertiary structure of human insulin with increasing surfactant concentration as shown by electronic circular dichroism. (TIF)

S3 Fig Changes in the tertiary structure of transferrin with increasing surfactant concentration as shown by electronic circular dichroism. (TIF)

S4 Fig Changes in the secondary structure of human insulin with increasing surfactant concentration as shown by electronic circular dichroism. (TIF)

S5 Fig Changes in the secondary structure of transferrin with increasing surfactant concentration as shown by electronic circular dichroism. (TIF)

S6 Fig Changes in the secondary structure of β-lactoglobulin with increasing surfactant concentration as shown by electronic circular dichroism. (TIF)

S7 Fig Absorbance as a function of time at a high flow rate, u = 30cm/s, in a L equals ∼30 m long capillary. The concentration distribution is shown for β-lactoglobulin-SDS system. The concentration of SDS is 4.45X10^-3^ M.
(TIF)

S8 Fig Absorbance as a function of time at a high flow rate, u = 30cm/s, in a L equals ∼30 m long capillary. The concentration distribution is shown for insulin-SDS system. The concentration of SDS is 1.30X10^-2^ M.
(TIF)

S9 Fig Absorbance as a function of time at a high flow rate, u = 30cm/s, in a L equals ∼30 m long capillary. The concentration distribution is shown for transferrin-SDS system. The concentration of SDS is 2.25X10^-4^ M.
(TIF)

S10 Fig Viscosity of SDS samples in the solvent in 25°C. (TIF)

S11 Fig Autocorrelation function g2(t) versus time t (μs) for solution of β-lactoglobulin in buffer with concentration 2.5 mg/ml for all angle range (40°, 60°, 80°, 90°, 120°, 150°). (TIF)

S12 Fig Autocorrelation function *g*_2_(*t*) versus time t (μs) and exponential fit for solution of β-lactoglobulin (A), transferrin (B) and human insulin (C) with SDS. The plot corresponds to the experiment for angle 90 degree.
(TIF)
