# Supplementary material for: Denaturation of proteins by surfactants studied by the Taylor dispersion analysis
Source: PLoS One. 2017 Apr 20;12(4):e0175838. doi: 10.1371/journal.pone.0175838 (PMC5398553; doi:10.1371/journal.pone.0175838)
Supplement: S1 Supporting Information — (DOCX) [file pone.0175838.s001.docx]

**Supporting Information**

**Electronic circular dichroism measurements**

In the Figures S1, S2 and S3 we show CD spectra presenting changes in the tertiary structure of studied proteins for bigger number of surfactant concentrations than was presented in the manuscript.





**Figure S1.** Changes in the tertiary structure of β-lactoglobulin with increasing surfactant concentration as shown by electronic circular dichroism.





**Figure S2.** Changes in the tertiary structure of human insulin with increasing surfactant concentration as shown by electronic circular dichroism.





**Figure S3.** Changes in the tertiary structure of transferrin with increasing surfactant concentration as shown by electronic circular dichroism.

Additionally, we show CD spectra in the range 190-260 nm and their analysis (Figures S4, S5 and S6). For estimation of the secondary structural composition the ECD spectra were submitted to the Jasco Secondary Structure Estimation (SSE) software based on the PCR method. The multivariate analysis allows us quantitatively approximate the α-helix, β-sheet and random coil contents. Due to the strong absorbance by the PBS buffer, the ECD signal were obtained up to 190 nm, so the range of 190-260 nm were taken into consideration.

For **Insulin** the helical content decreases from 38% (native protein) to 28% when concentration of SDS reached 2.5 mg/ml, and the same time the β-structure increases from 18% to 27% in the same concentration range.

For **Transferrin** the helical content increases from 23% (native protein) to 32% when concentration of SDS reached 1.3 mg/ml whilst the decrease of β-structure from 37% (native protein) to 17% when concentration of SDS reached 1.3 mg/ml is observed.

For **β -lactoglobulin** the helical content increases noticeably from 12% (native protein) to 40% when concentration of SDS reached 2.5 mg/ml, and the decreases β-structure from 43% to 12% in the same concentration range.

Table S1. Summary of ECD experiments for proteins under investigation.

| Protein | Minimum SDS concentration [M]  which introduced substantial changes in protein structure | |
| --- | --- | --- |
|  | Based on II-structure region | Based on III-structure region |
| Insulin | 2.3 x 10^-4^ M | 2.3 x 10^-4^ M |
| Transferrin | 4.3 x 10^-4^ M | 4.3 x 10^-4^ M |
| β-lactoglobulin | 4.3 x 10^-4^ M | 4.3 x 10^-4^ M |





**Figure S4.** Changes in the secondary structure of human insulin with increasing surfactant concentration as shown by electronic circular dichroism.





**Figure S5.** Changes in the secondary structure of transferrin with increasing surfactant concentration as shown by electronic circular dichroism.





**Figure S6.** Changes in the secondary structure of β-lactoglobulin with increasing surfactant concentration as shown by electronic circular dichroism.

**Taylor Dispersion Analysis at high flow rates**





**Figure S7.** Absorbance as a function of time at a high flow rate, u = 30cm/s, in a L equals ∼30 m long capillary. The concentration distribution is shown for β-lactoglobulin-SDS system. The concentration of SDS is $4.45 \times{10}^{-3}$M.

**

**

**Figure S8.** Absorbance as a function of time at a high flow rate, u = 30cm/s, in a L equals ∼30 m long capillary. The concentration distribution is shown for insulin-SDS system. The concentration of SDS is $1.30 \times{10}^{-2}$M.





**Figure S9.** Absorbance as a function of time at a high flow rate, u = 30cm/s, in a L equals ∼30 m long capillary. The concentration distribution is shown for transferrin-SDS system. The concentration of SDS is $2.25\times{10}^{-4}$M.

**Viscosity of samples**

The viscosity experiments were performed to check whether the micelles containing SDS have influence to dynamic viscosity of samples. All the tests were perform in 25 Celsius degree, using cone-plate geometry with diameter 50 mm and angle 1 degree, by the Rotational Rheometer Kinexus Pro produced by Malvern Instruments LTD.

During the measurements the samples with concentration lower and higher than CMS (1.29 mg/ml) were tested. In Figure S10 the results are shown with the comparison of water viscosity.

According to those tests we assumed that viscosity of our samples is constant and it is equal to the water viscosity 0.89 mPa s.





**Figure S10.** Viscosity of SDS samples in the solvent in 25 °C.

**Dynamic light scattering**

The DLS measurements were conducted in the angle range from 40 to 150 degree for all samples. In Figure S11 we show the example for β-lactoglobulin in buffer solution with an indication of changing of the decay time with angle.





**Figure S11.** Autocorrelation function g2(t) versus time t (μs) for solution of β-lactoglobulin in buffer with concentration 2.5 mg/ml for all angle range (40⁰, 60⁰, 80⁰, 90⁰, 120⁰, 150⁰).

Autocorrelation function g_2_(t) was fitted using double-exponential function

$g_{2}\left( t \right) = {(A\cdot exp(-t/B)+C\cdot exp(-t/D))}^{2}+E$ (S1)

where A,B,C,D and E are fitted parameters (Figure A and B in S12).

Data for the insulin-SDS system were fitted using mono-exponential fit (Figure C in S12).


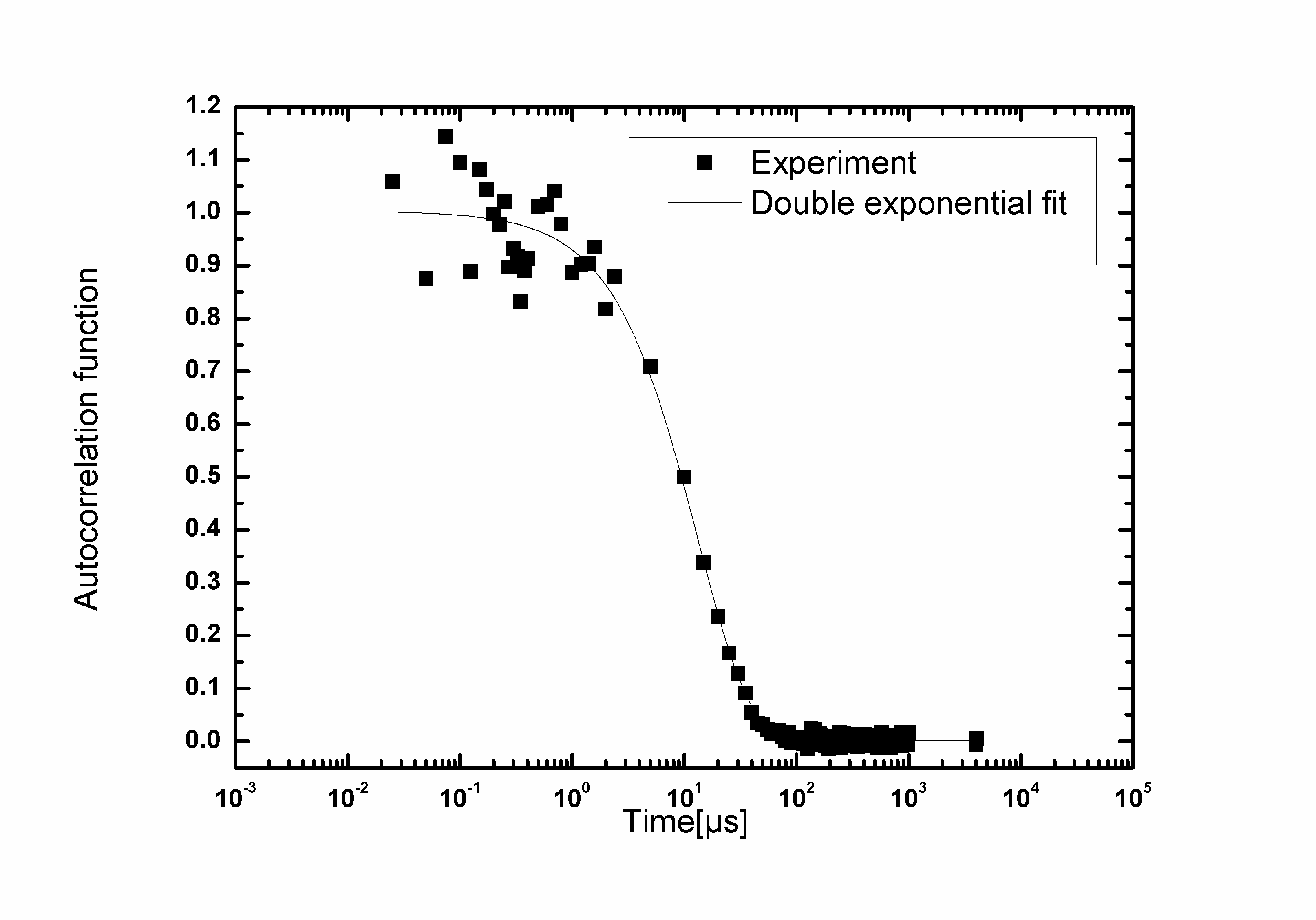


**A**


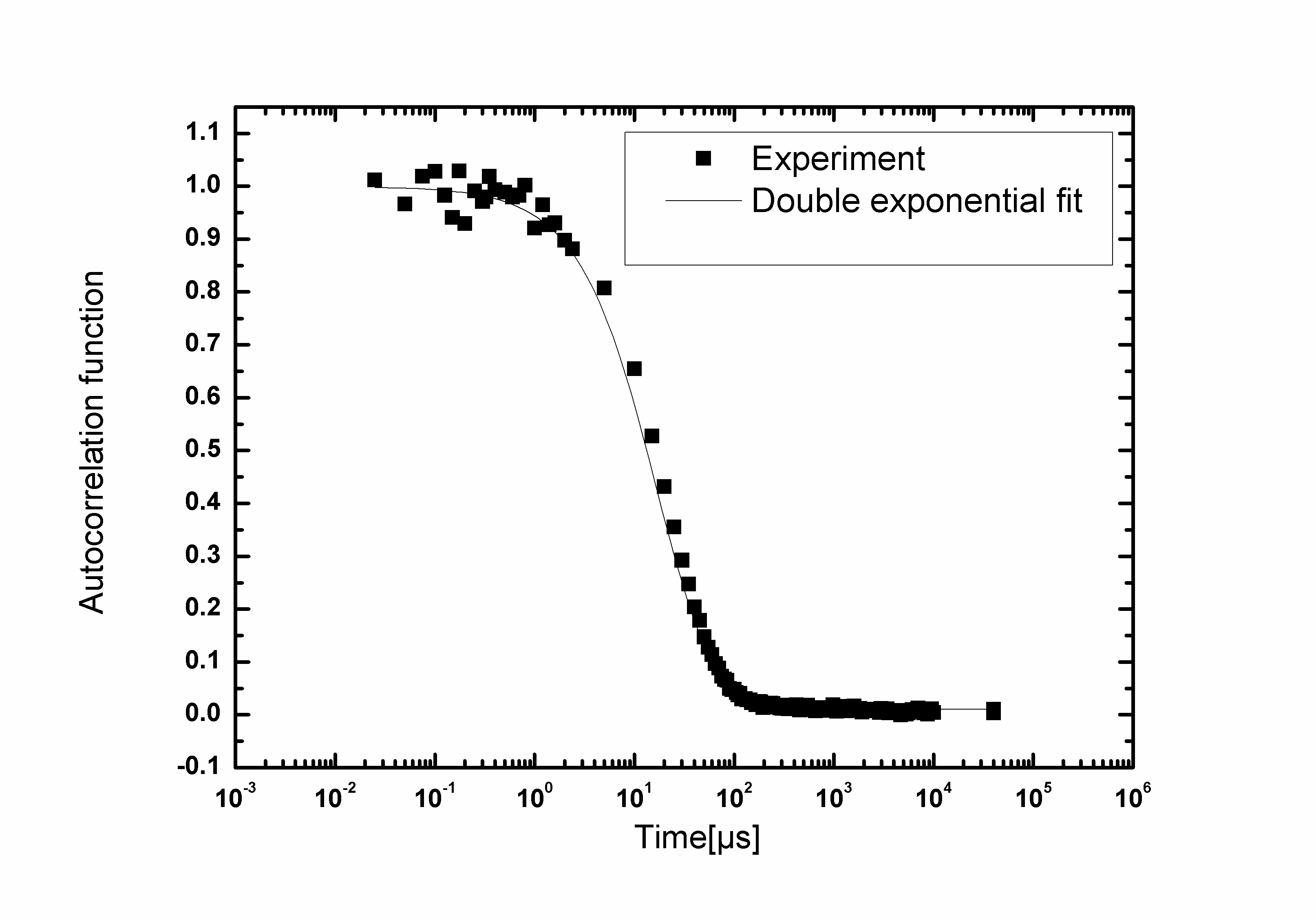

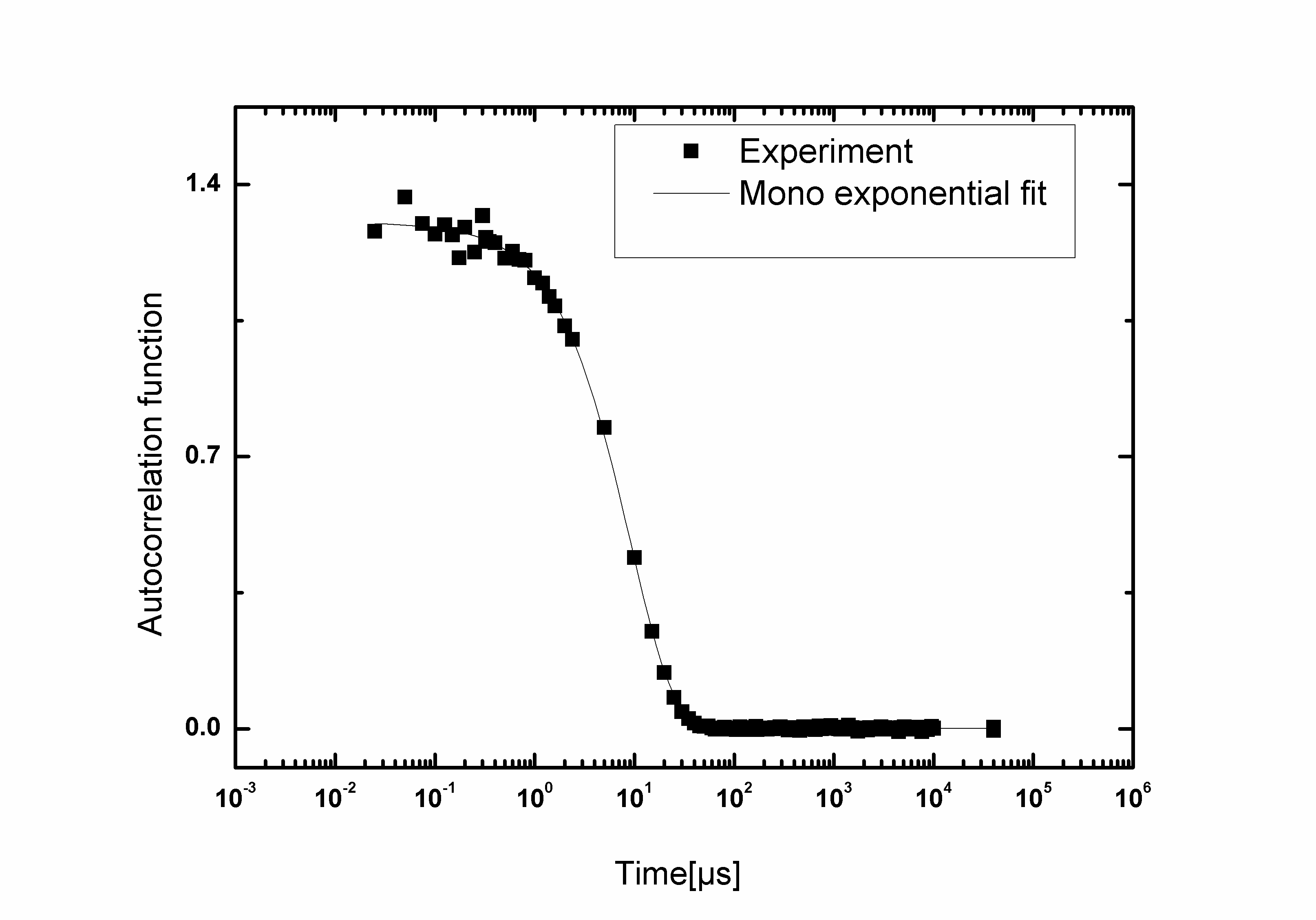


**B**

**C**

**Figure S12.** Autocorrelation function $g_{2}\left( t \right)$ versus time t (μs) and exponential fit for solution of β-lactoglobulin (A), transferrin (B) and human insulin (C) with SDS. The plot corresponds to the experiment for angle 90 degree.
