# Supplementary figures and images for: Denaturation of proteins by surfactants studied by the Taylor dispersion analysis
Source: PLoS One. 2017 Apr 20;12(4):e0175838. doi: 10.1371/journal.pone.0175838 (PMC5398553; doi:10.1371/journal.pone.0175838)

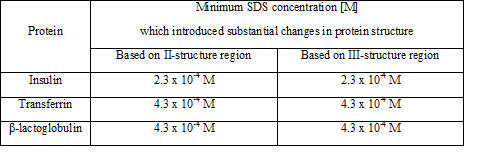

Supplement: S1 Table — (TIF) [file pone.0175838.s002.tif]

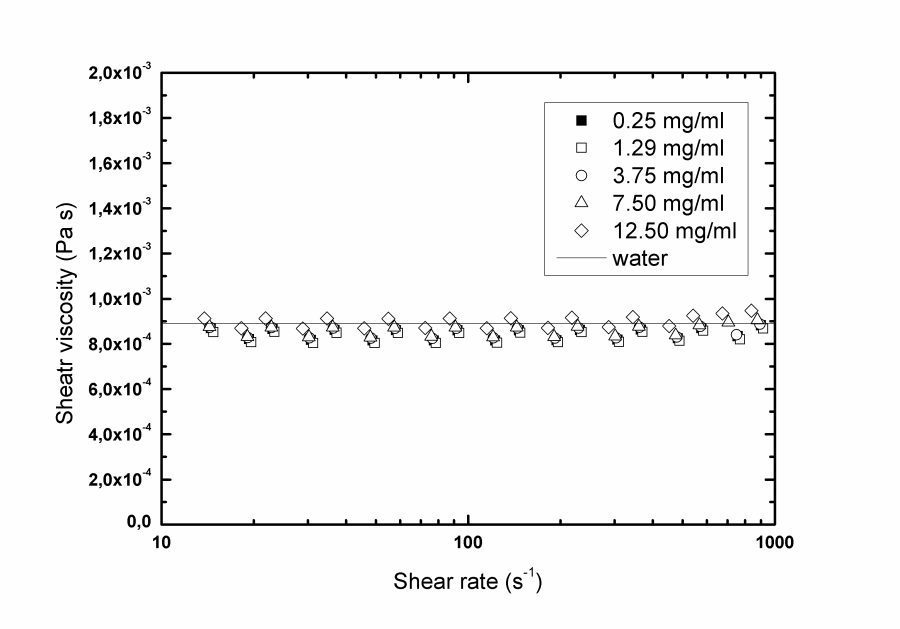

Supplement: S10 Fig — (TIF) [file pone.0175838.s012.tif]

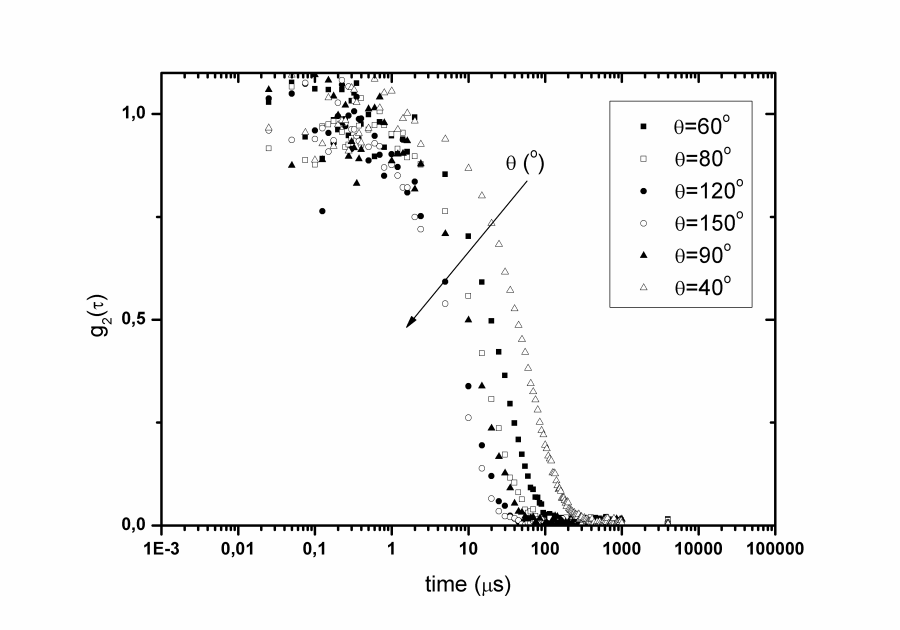

Supplement: S11 Fig — (TIF) [file pone.0175838.s013.tif]

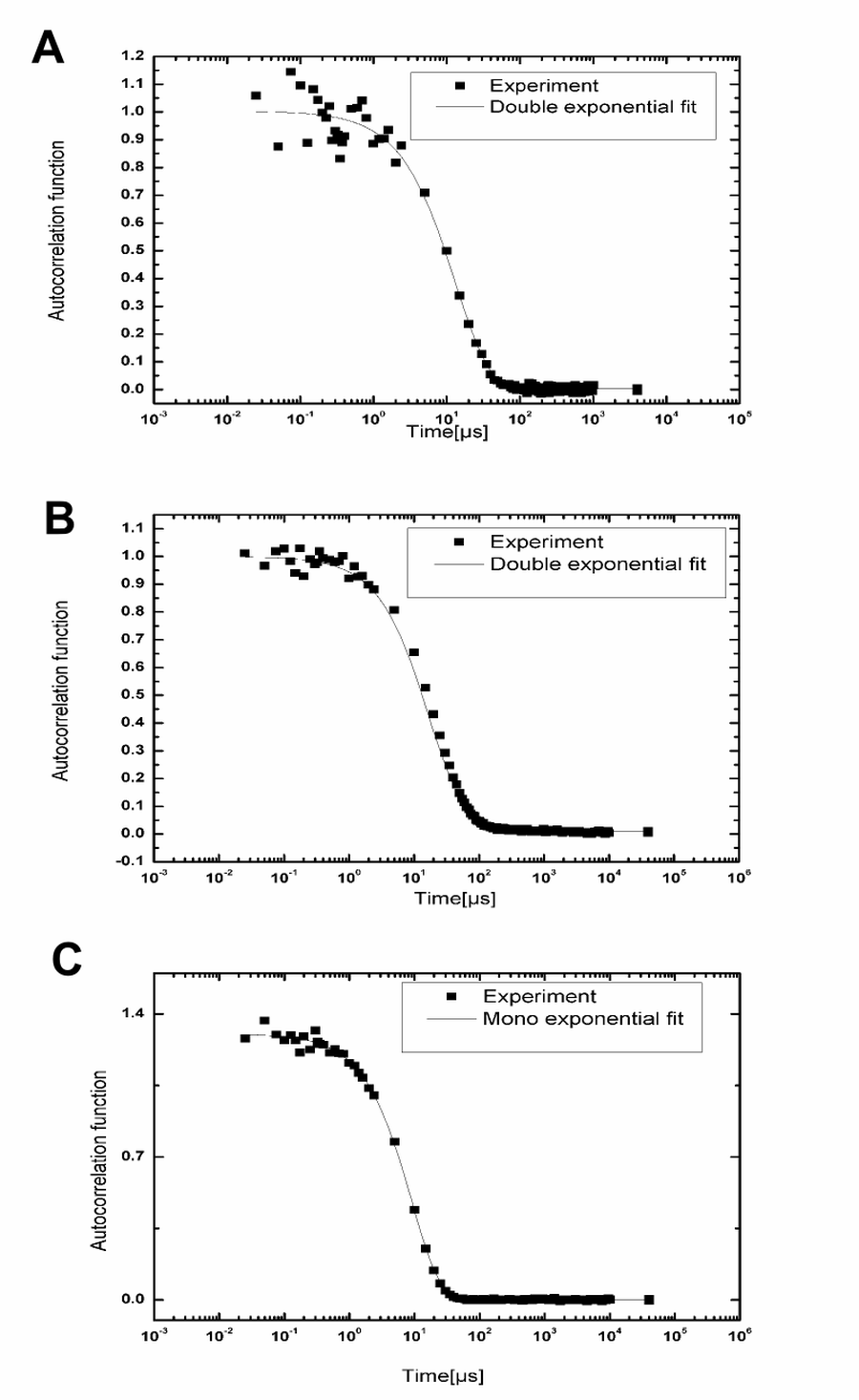

Supplement: S12 Fig — The plot corresponds to the experiment for angle 90 degree. (TIF) [file pone.0175838.s014.tif]
